# Exploring the Influence of Social Infrastructure on Experiences of Aging in Place in Dublin, Ireland

**DOI:** 10.1093/geroni/igaa057.2389

**Published:** 2020-12-16

**Authors:** Hannah Grove

**Affiliations:** Maynooth University, Maynooth, Kildare, Ireland

## Abstract

This paper presents results from a PhD project that has carried out interviews, mapping exercises and ‘go-along’ interviews with thirty-four people aged sixty-six and over, in an inner city and suburban study area within Dublin, Ireland. A key objective of this research was to identify places, activities and interactions of most importance to older people. Through qualitative and spatial data collection and analysis techniques, different types of ‘social infrastructure’ that individuals value and engage with, as part of their daily lives, are identified and presented using annotated maps. The interactions and types of activities that occur in these places, and why they are important, are also discussed. Results demonstrate the benefits of high quality social infrastructure (and the challenges associated with a lack of social infrastructure) through participant’s own experiences, and highlight the vital role this plays in supporting older people to age well in place.

